# Measuring, modeling and fostering embodiment of robotic prosthesis

**DOI:** 10.3389/fnrgo.2024.1400868

**Published:** 2024-05-21

**Authors:** Adna Bliek, Daniel Andreas, Philipp Beckerle, Tim Rohe

**Affiliations:** ^1^Chair of Autonomous Systems and Mechatronics, Department of Electrical Engineering, Faculty of Engineering, Friedrich-Alexander-Universität Erlangen-Nürnberg, Erlangen, Germany; ^2^Department Artificial Intelligence in Biomedical Engineering, Friedrich-Alexander-Universität Erlangen-Nürnberg, Erlangen, Germany; ^3^Institute of Psychology, Friedrich-Alexander-Universität Erlangen-Nürnberg, Erlangen, Germany

**Keywords:** rubber hand illusion, rubber limb illusion, cognitive modeling, multisensory causal inference, multisensory integration, robotic prosthesis, robotic limb illusion

## 1 Introduction

Amputees can be fitted with a robotic prosthesis to restore the function and appearance of the missing limb. Integration of the prosthesis into the body schema based on visual, proprioceptive, and haptic signals is assumed to be a prerequisite for effective use. In the ideal case, a prosthesis user could operate a robotic limb as well as a natural limb and would have a similar feeling of ownership and agency, i.e., perceived control, of the prosthetic limb, leading to a high acceptance (Makin et al., 2017). In other words, the user may fully embody the prosthesis (Makin et al., 2017), which has been shown to reduce phantom limb pain (Dietrich et al., 2012; Svensson et al., 2017). Technical innovations aim at fostering the integration of the robotic prosthesis, e.g., by providing haptic feedback (Antfolk et al., 2012; Huynh et al., 2019). Currently, this ideal case has not been reached yet and users often still lack acceptance of their prosthesis (Salminger et al., 2022). To design embodied and accepted prostheses, we believe that an interdisciplinary three-staged framework is needed, measure-model-foster: First, an ecologically valid experimental paradigm to measure bodily integration of robotic prostheses. Second, a computational model to understand the underlying neurocognitive mechanisms. Third, technical innovations that use these findings to foster embodiment of prostheses.

## 2 A framework for embodiment of robotic prostheses

### 2.1 Measuring robotic limb embodiment

A promising experimental paradigm to measure embodiment is the “rubber hand illusion” (RHI), a well-established multisensory illusion to investigate own-body perception (Botvinick and Cohen, 1998). In the RHI, humans perceive ownership of a rubber hand close to their own hidden hand if both rubber and own hand are tactically stroked in synchrony for 7–80 s (Lloyd, 2007; Rohde et al., 2011). RHI is based on multisensory perception, as the participants integrate the haptic and proprioceptive signals of their own hand with the visual signals of the rubber hand. The integration of the rubber hand into the own body schema can be measured implicitly via a proprioceptive drift of the felt position of the own hand toward the rubber hand, as well as explicitly by subjective ownership ratings. More generally, not only a hand but also rubber limbs such as a foot can be integrated to create the “rubber limb illusion” (RLI) (Flögel et al., 2016). Recent studies confirmed that a robotic limb is integrated into the body schema under similar conditions as a rubber limb, thus establishing the experimental paradigm of the “robotic limb illusion” (RobLI) (Caspar et al., 2015; Romano et al., 2015; Huynh et al., 2019). When participants actively move the robotic limb by own-limb movements, e.g., using a sensor glove, the feelings of ownership and proprioceptive drift can be induced comparably to a long passive haptic stimulation in the RHI (Riemer et al., 2019). Additionally, active movement was found to have a positive influence on agency compared to passive movement during the RHI (Kalckert and Ehrsson, 2014). Thus, the RobLI paradigm allows to reliably measure whether and how a robotic limb is integrated into the body schema. Beyond an experimental paradigm to measure the embodiment of a prosthesis, researchers need a conceptual and computational model to describe, explain, and predict how neurocognitive mechanisms elicit the RobLI. A neurocognitive model further allows to predict how specific stimuli modulate embodiment. Such modulating factors help to design prosthesis features that foster embodiment, e.g., via sensory feedback or even bidirectional communication and adaptation between user and prosthesis (Beckerle et al., 2019).

### 2.2 Modeling multisensory integration

**Figure 1:**
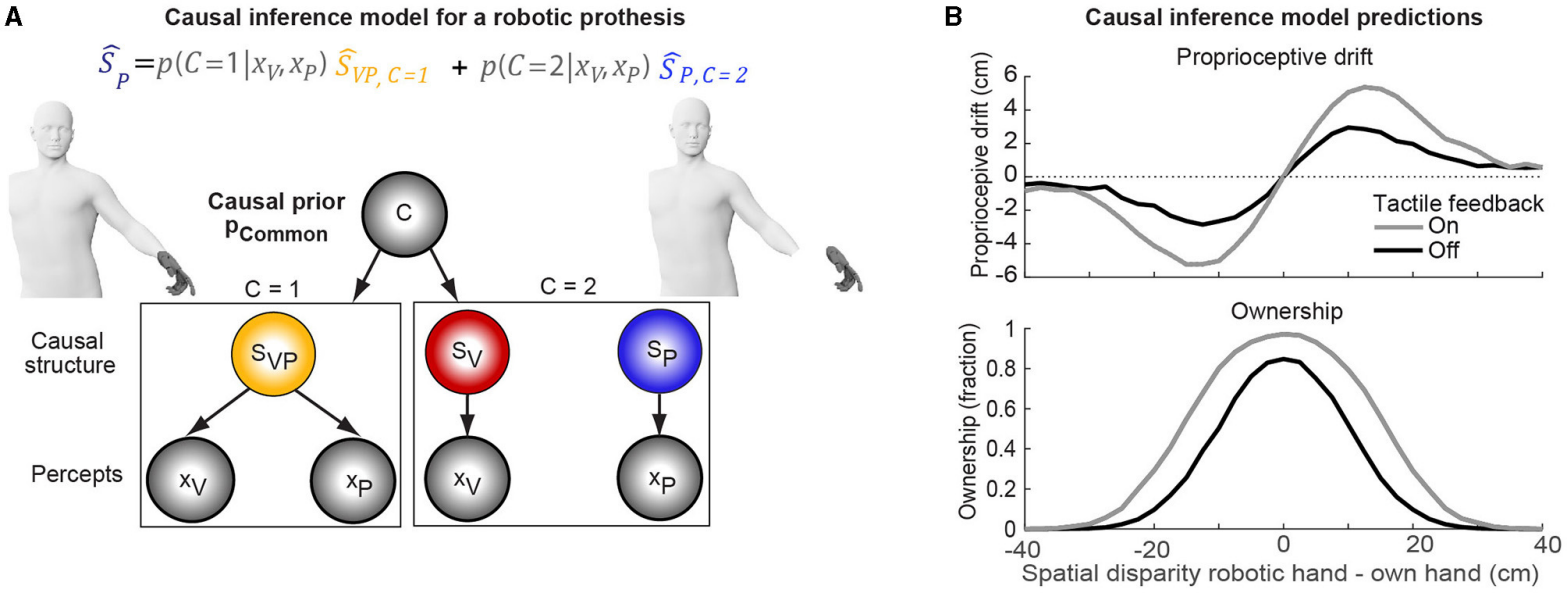
Causal inference (CI) model and its predictions for the robotic hand illusion as an example of the robotic limb illusion. **(A)** The CI model assumes that users infer from the sight of the robotic hand (*x*_*V*_) and the proprioceptive feeling of the own hand (*x*_*P*_) whether both signals arise from the same cause—the robotic hand. When users infer a common cause, they incorporate the robotic hand into their body schema, thus reporting ownership of the hand [i.e., quantified by *p*(*C* = 1|*x*_*V*_, *x*_*P*_)], and a proprioceptive drift (i.e., quantified by Ŝ_*P*_). If users infer independent causes, the robotic hand feels alien (i.e., users do not report ownership or proprioceptive drift). If the signals' causal structure is uncertain, i.e., neither a common nor independent cause is unequivocal, users only partially embody the robotic hand. **(B)** The CI model's predictions for ownership and proprioceptive drift depend on the spatiotemporal disparity (i.e., misalignment) of the own and the robotic hand in a RobLI experiment: e.g., for a low spatial disparity, the CI model predicts high ownership ratings and high proprioceptive drift which both decrease at larger disparities suggesting independent causes. Haptic feedback may increase embodiment of a robotic hand as indicated by a widened spatiotemporal integration window. In the formal CI model, haptic feedback would be implemented as a third haptic percept. Note that the panel shows the CI model's schematic predictions. Human figure adapted from © Mihai Zaharia/Adobe Stock.

The Bayesian causal inference (CI) model of multisensory perception (Körding et al., 2007; Samad et al., 2015; Shams and Beierholm, 2022) establishes a general conceptual and computational approach to understand and investigate why participants integrate an external robotic limb into their own body schema or refrain from doing so. The CI model assumes that the brain needs to probabilistically infer the causal structure of multisensory signals if it seeks to integrate the signals from a common cause or to segregate the signals from independent causes (Figure 1A). For the RobLI, the CI model makes quantitative predictions about why and how the brain integrates visual-proprioceptive signals to create the illusion: The brain computes the likely causal structure of proprioceptive signals from the residual limb and the visual signal of the prosthesis by combining the prior assumption that the robotic limb belongs to the own body with information on the signals' spatiotemporal relations (Wallace et al., 2004; Rohe and Noppeney, 2015a,b; Rohe et al., 2019). If users infer that the robotic limb is the common cause of the signals, they explicitly perceive ownership of the robotic limb and optimally integrate the own limb's and robotic limb's signals by weighting them proportionally to their relative sensory noise (Ernst and Banks, 2002; Alais and Burr, 2004). Thus, the integration elicits an unconscious proprioceptive drift of the own limb's felt position toward the robotic limb. If users infer independent causes, they feel the robotic limb as alien and segregate the signals (Wallace et al., 2004; Rohe and Noppeney, 2015a). The Bayesian CI model can thus be used to predict whether and how users integrate a robotic limb into their body schema: The model predicts that the inference of a common cause depends on visual-proprioceptive sensory noise as well as the perceived spatial and temporal alignment of the own proprioceptive information and the visual signal of the robotic limb, leading to a spatiotemporal integration window as a key profile (Figure 1B).

Indeed, previous studies showed that the RLI depends on stimulus factors such as temporal disparity, synchronicity of the visual-haptic signals, or sensory noise. In the first study on the RLI, Botvinick and Cohen (1998) describe that small asynchronies between the real and rubber hand stimulation resulted in a significant reduction in the experience of the illusion. Subsequent studies showed that the RLI only diminishes strongly for delays of 300 ms and larger (Bekrater-Bodmann et al., 2014). The robustness of the RLI against small temporal disparities can be very useful when considering robotic limbs, as there will always be small time delays in movement due to the computation time and the time it takes to send the commands to the robotic limb. Another well-researched factor is the spatial disparity. The maximum displacement to create an illusion depends on the direction of the displacement. For horizontal displacements, a gradual decrease was found for the RHI between 15 and 67.5 cm (Lloyd, 2007). When using the RobLI to investigate the embodiment of prosthetic hands, it is important to note that the peripersonal space of the amputee reduces over time (Canzoneri et al., 2013). This adapted peripersonal space might lead to a reduced embodiment of the prosthesis. However, small displacements of the limb do not abolish the RLI and should thus not strongly compromise the embodiment of a prosthesis. Beyond spatiotemporal factors, sensory noise modulates the RLI. In recent studies, the effect of visual and proprioceptive noise on the RLI was investigated (Chancel et al., 2022a; Chancel and Ehrsson, 2023). In both studies, the induction of the RLI increased with the amount of noise that was used. Recent studies applied the CI model to accurately predict how spatiotemporal factors modulate proprioceptive drift and ownership in the RLI (Samad et al., 2015; Fang et al., 2019; Chancel et al., 2022a,b). While the CI model was successful in predicting proprioceptive drift for single positions of the own and an artificial limb (Schürmann et al., 2019), the CI key prediction of a spatiotemporal integration window for the RobLI (Figure 1B) still awaits empirical validation.

Overall, these studies suggest that the spatiotemporal alignment of signals from own limbs and robotic limbs is critical to foster the embodiment of a robotic prosthesis because spatiotemporal alignment leads the brain to infer a common cause of the signals—the own body. The CI model's predicted spatiotemporal integration windows have not yet been experimentally investigated for the RobLI, but such a prediction is supported by the factors influencing the RLI. Furthermore, technical innovations such as haptic feedback might foster the user's inference of a common signal cause and, therefore, lead the user to integrate the prosthesis into the body scheme with higher acceptance.

### 2.3 Fostering embodiment by user feedback

The addition of spatiotemporally aligned visual, auditory, and/or haptic sensory feedback from the prosthesis to the user will increase embodiment of the prosthesis according to the CI model. Haptic feedback is a common method to inform the user of their current interactions with various devices and applications. It can inform the user regarding the dynamics of the interaction as compared to just the kinematic information obtained using visual feedback, e.g., increasing grasping forces with a prosthetic hand can be conveyed using an increasing intensity of vibrotactile feedback. In terms of the CI model, haptic feedback may provide additional sensory evidence that leads the user to infer a common cause and to embody the prosthesis (Figure 1B): Spatiotemporally aligned haptic feedback would provide the brain with consistent trisensory visuo-proprioceptice-haptic information that the prothesis belongs to the same cause, that is the own body.

A study by Huynh et al. (2019) suggests that visuotactile and visuomotor feedback equally contribute to increase embodiment. A combination of both can lead to even better results, which shows the importance of implementing haptic feedback in prosthetic devices. Haptic feedback can be divided into two major categories, which are invasive and non-invasive methods. Invasive methods such as peripheral/central nervous system stimulation, and targeted sensory reinnervation can provide more intuitive feedback by providing a more accurate stimulation and a more natural feel (Svensson et al., 2017). However, those methods impose an additional risk by the surgical procedure and in some cases suffer from short implant lifespans. Haptic feedback methods such as mechanotactile, vibrotactile, and electrotactile signals are often noisy and not somatotopically matched, so users need to learn the mapping of the feedback locations to the lost fingers. We believe that to date, non-invasive techniques are the preferred option due to their lower risk and easier implementation. Haptic feedback has to ensure that stimulations on the location of the residual limb feel like stimulations on the amputated hand. To achieve this somatotopic match, the location of the haptic stimulus should ideally match the map of referred sensations on the residual limb (Björkman et al., 2016; Svensson et al., 2017). Thus, the users need to learn the mapping of the feedback locations to the lost fingers (Svensson et al., 2017). The map of referred sensations differs between amputees with some individuals showing either only a limited map or no map at all (Björkman et al., 2016), which prevents its use for haptic feedback. Using the map can be further complicated by a small size of the residual limb and technical reasons such as space limitations in the prosthesis shaft or interference with sensors to control the prosthesis. Thus, future studies need to compare different feedback locations for non-invasive haptic feedback. Critically, we believe that the RobLI experimental paradigm and the CI model will allow to measure and model how spatiotemporal design features of haptic feedback influence the embodiment of a prosthesis. Additionally, such experiments could be used to improve the design of future haptic feedback devices concerning the used modality, intensity mapping, or even aesthetic and comfort to further increase embodiment. If the CI model is computationally and experimentally fully established to describe and predict the embodiment of robotic prostheses, it would also allow to directly simulate the psychological effects of feedback design choices and thereby potentially shorten development cycles.

## 3 Discussion

Designers of robotic prostheses need to consider how multisensory neurocognitive processes shape the integration of a prosthesis into the body schema. The CI model makes strong predictions for optimal design choices that will enable the user to feel ownership, agency, and acceptance of a prosthesis. The model predicts that many factors such as spatiotemporal disparities and haptic feedback influence the integration of robotic limbs into one's body schema. Yet so far, the CI model has been only rarely applied to describe the embodiment of robotic prostheses as compared to the rubber limb illusions (Samad et al., 2015; Schürmann et al., 2019). The critical next step will be to validate the model for the RobLI by systematically manipulating factors that influence the causal inference process, such as spatiotemporal alignment (Figure 1B) and haptic feedback. With the addition of haptic feedback, for instance, we expect to see an increase of the user's spatiotemporal integration window and the overall ownership rating. In an experiment with active object-targeting motion that provides haptic feedback from the robotic limb, the inference of a common cause may also lead to a higher perceived agency over the robotic limb.

In conclusion, we propose a three-stage framework to achieve embodied robotic prosthesis: RobLI experiments measure how spatiotemporal factors modulate ownership, agency, and proprioceptive drift, multisensory causal inference quantitatively models the factors' influence on embodiment, and haptic feedback fosters the users' inference that the robotic hand is their own. Critically, the framework allows to recursively and interactively optimize robotic prostheses including sensory feedback in a user-centered design. This challenging framework requires interdisciplinary methods and collaborations from medical engineering, neurocognitive psychology, and computer science. Yet, in our opinion, only this framework will bridge the gap between the technical challenges of prosthesis design and the psychological challenges of making a user feel and use a prosthesis as part of their own body.

## Author contributions

AB: Conceptualization, Writing – original draft, Writing – review & editing. DA: Conceptualization, Writing – original draft, Writing – review & editing. PB: Conceptualization, Writing – original draft, Writing – review & editing. TR: Conceptualization, Writing – original draft, Writing – review & editing.
